# The representation of adolescent social media use: a systematic review and content analysis of UK newspaper articles

**DOI:** 10.1186/s12889-025-23897-5

**Published:** 2025-09-24

**Authors:** Jade Davies, Sean Cadwallader, Louise Black, Jo Hickman Dunne, Margarita Panayiotou

**Affiliations:** 1 Manchester Institute of Education, School of Environment, Education and Development, University of Manchester, Manchester, UK; 2 School of Social Sciences, University of Manchester, Manchester, UK

**Keywords:** Social media, Adolescent, Mass media, Risk factors, Mental health

## Abstract

**Background:**

The rapid rise in social media use among adolescents has led to widespread public and academic debate about its potential risks, especially concerning mental health. Media representations are integral in shaping public and policy attitudes towards such issues. This study therefore critically examines how UK newspapers portray the risks and benefits of adolescent social media use.

**Methods:**

We systematically searched for articles discussing adolescent social media use published in UK newspapers or on the BBC News website between 2014 and 2024. In total, 307 met our criteria for inclusion. We conducted a content analysis of the included articles, paying attention to the specific risks and benefits discussed, the sources of information used, and the sentiment of the headlines. We used cross-tabulation analyses to examine how representations varied across different newspapers, analysing associations between publication type, use of research evidence and quotations, and reporting of risks/benefits.

**Results:**

Most headlines (58%) were negatively framed, with tabloids generally adopting a more negative tone than broadsheets. The overwhelming majority of articles (98%) discussed potential risks, with mental health concerns, exposure to harmful content, online conflict, and inappropriate adult contact being the most frequently cited concerns. Almost one in every four articles (24%) discussed suicidality and/or suicide in relation to adolescent social media use. In contrast, only 16% of articles discussed any potential benefits of adolescent social media use. Fewer than one-third of articles (31%) used evidence to support claims. When evidence was included, grey literature, such as reports from charities or government bodies, was used more than twice as often as academic research.

**Conclusions:**

Our findings highlight a disproportionate focus on the potential risks of adolescent social media use relative to the potential benefits, especially regarding mental health. This framing is somewhat inconsistent with the current empirical evidence and may contribute to increased public anxiety. This framing also has the potential to shape policy agendas in ways that do not fully reflect young people’s lived experiences. We call for more balanced, evidence-based media coverage to foster a more informed public conversation. Improving the accuracy and sensitivity of reporting could, in turn, support the development of more effective policy responses and interventions.

## Introduction

More young people are using social media now than ever before. In the UK, social media usage among eight to 11-year-olds rose from 18% in 2018 to 63% in 2022, while usage among 12- to 15-year-olds increased from 69 to 93% over the same period [85, 86]. The impact of this digital transformation is complex, with research pointing toward both potential benefits and risks. For example, some research suggests these platforms offer young people opportunities to connect and communicate with family and friends, access information, and seek entertainment in ways that previous generations could not [9, 49, 64, 99, 109, 127]. At the same time, studies have identified potential negative experiences such as cyberbullying, sexting, and exposure to inappropriate content [67, 112]. Research examining the relationship between social media use and adolescent mental health reveals a similarly mixed picture, with some studies finding links to worsening mental health (e.g., depression, anxiety or wellbeing), and others finding no significant relationship, or even potentially the reverse relationship [21, 56, 91, 111, 115, 121, 131].

The rapid proliferation of research in this field has outpaced methodological rigour, with many studies suffering from small sample sizes and an overreliance on cross-sectional designs, resulting in difficulties in establishing causal relationships [89]. As a result, current understanding of the overall *consequences* of social media use on adolescents remains tentative. Despite the mixed evidence base and research limitations, public perception appears to be characterised by widespread concern about the potential psychological harm associated with adolescent social media use [89]. Indeed, in a survey asking UK adults to select the most important reasons for the increase in adolescent mental health problems, 65% selected the increased use of social media [31].

### The role of mass media in shaping panic

Concerns regarding the potential harmful effects of social media use on adolescent development reflect a long-standing pattern of public anxiety surrounding emerging technologies. Throughout history, technological advances have consistently sparked corresponding waves of concern, from fears of ‘reading addiction’ in the eighteenth century to fears of ‘radio addiction’ in the 1940's, and later concerns about television and games [88]. These reactions may be conceptualised as moral panics [17], in which certain individuals, groups, or practices – in this case, new technologies – are perceived as significant threats to societal norms, values, and social order. Central to moral panic theory [17] is the role of mass media (e.g., television, newspapers), which is posited to amplify public fear by sensationalising issues and prompting responses from policymakers and ‘moral entrepreneurs’, which further reinforce a sense of crisis.

The belief that media can influence societal attitudes and behaviours is long held. A central mechanism through which this influence may occur is the use of media narratives. Media narratives are stories or framings that take place in time and serve as a means through which we (the audience) learn about the world and ourselves [4]. Through the repetition of narratives, media outlets can signal which topics are most important, effectively setting the public agenda [75, 76]. The way these narratives frame issues – whether positively or negatively – can then shape how they are understood and interpreted [34]. Over time, persistent exposure to the same media narratives can lead to their widespread acceptance as reality [44], and result in the adoption of new attitudes, values, and behaviours [2].

These media effects are, however, not uniform. For example, The Differential Susceptibility to Media Effects Model [118] suggests that media impact varies based on existing dispositional, developmental, and social susceptibilities. Audiences are also increasingly recognised as active participants in interpreting media messages. For example, Livingstone [66] argues that audiences are not “gullible, homogenous, and unthinking” (p.172) but that they actively interpret, negotiate, and sometimes resist media content based on their own experiences and social positions. Thus, media has the potential reinforce existing beliefs as well as shape new ones. Gavin’s [43] review of media effects on British politics lends credence to these ideas, concluding that mass media play a powerful role in shaping public attitudes toward highly salient matters (e.g., national immigration, climate change) through both attitude formation and reinforcement. It is therefore plausible that the widespread anxiety about adolescent social media use, is, at least in part, shaped by media reporting on the issue.

### The potential impact of media panic

The way media portray adolescent social media use is important, not just for public opinion, but for adolescents’ lived experiences. According to Bronfenbrenner’s [15] Ecological Systems Theory, adolescent development is shaped by four key systems: the microsystem which includes close, direct influences (e.g., parents, peers, teachers), the mesosystem which includes connections between environments (e.g., interactions between parents and teachers), the exosystem which includes indirect environments (e.g., local government), and finally the macrosystem, which encompasses broader cultural norms and societal and values.

**Fig. 1:**
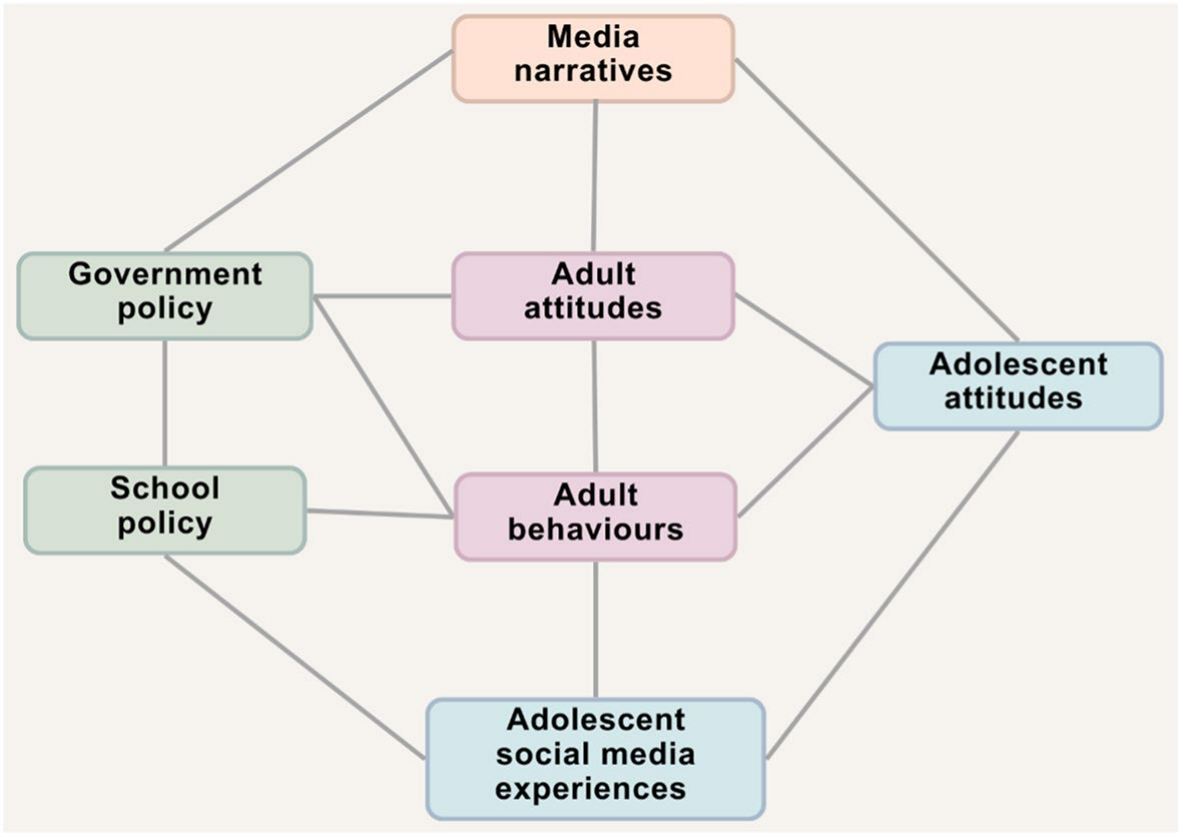
Visual representation of potential pathways between media narratives and adolescent social media experiences

Mass media reside within the exosystem, influencing the cultural narratives adolescents encounter and, subsequently, their experiences. For example, if adolescents repeatedly encounter narratives portraying social media as inherently harmful, they may internalise these messages, a mindset associated with poorer psychological wellbeing [62]. Neal and Neal's [77] networked model extends Ecological Systems Theory by highlighting the dynamic interplay between systems. In this model, mass media are positioned not as passive broadcasters but as dynamic agents that interact with other ecological systems. For example, media narratives about social media risks (which occur within the exosystem) may shape parents' digital supervision practices (which occur within the microsystem), ultimately affecting adolescents'online experiences. See Fig. 1 for a visual representation of potential pathways between media narratives regarding adolescent social media use and adolescent experiences.

Media narratives may also influence policymakers, impacting the political agenda (in the macrosystem) and driving tangible policy change [57, 119, 122]. This was demonstrated in the case of the ‘Blue Planet Effect’ where media coverage on plastic pollution during the BBC documentary series *Blue Planet II* resulted in key policy shifts [104]. Indeed, Google searches for “ocean plastic” significantly increased after the series aired, and the series and/or its host were referenced in 11 of 41 subsequent government discussions, prompting a series of policy changes [26–28, 71]. While this example demonstrates the potential for media to drive *positive* government action, the potential for policy interventions based on predominantly negative media narratives regarding adolescent social media use is concerning, especially when the research evidence remains fundamentally inconclusive. Indeed, while targeted regulations based on such reporting might aim to protect young people, hasty or overly restrictive policies – including blanket social media bans for adolescents (e.g., [95]) – could inadvertently limit the potential positive aspects of digital connectivity, potentially constraining adolescents' social experiences and opportunities for digital literacy and social interaction.

### Newspapers as a key source of information

Since their emergence in the early seventeenth century, newspapers have played a pivotal role in public information dissemination. Initially catering to a literate elite, their reach expanded dramatically in the nineteenth century with advances in printing technology, which enabled mass circulation and prompted the diversification of content [129]. The rise of the tabloid press in the 1970 s further transformed the landscape, with these papers using accessible language and human-interest stories to engage a broader, more diverse readership [129]. The pursuit of mass readership also introduced sensationalist reporting practices, with publishers recognising that dramatic headlines and scandalous coverage was effective in boosting circulation and thus revenue [55]. This style of reporting prioritises emotional impact over factual accuracy, often amplifying negative events to capture public attention [107]. Contemporary research demonstrates that these sensationalist tendencies persist (e.g., [14]), and, while once confined to the tabloid press, there are growing concerns of a broader 'tabloidisation' across the entire news media landscape [18, 92].

Newspaper readership in the UK reached its peak in the early 1950 s, with around 85% of the population reportedly reading a paper every day, more than any other nation [7]. The subsequent rise of internet has, however, fundamentally challenged the traditional newspaper industry, leading to declines in readership as audiences have moved to online platforms that offer instant, free access to news content [40, 82]. While newspapers have responded by launching digital editions, mobile applications, and online subscription models, these adaptation efforts have achieved limited success in attracting younger generations, who increasingly bypass newspapers entirely in favour of consuming news through alternative means (e.g., social media) [87].

Nevertheless, newspapers continue to represent a key source of information for a substantial proportion of the UK adult population [87]. Indeed, most people perceive newspapers as high-quality, accurate, and trustworthy [87], demonstrating the continued importance of their influence. Moreover, newspaper content continues to shape wider public discourse by being shared, summarised, or cited across social media platforms, thereby influencing audiences who may not engage with newspapers directly [54]. This enduring presence highlights the continued importance of newspapers in informing civic life and setting the broader media agenda, and points toward the need to understand how newspapers represent adolescent social media use.

### Newspaper representations of adolescent social media use

Emerging research points toward a panicked media discourse surrounding adolescent social media use. Stern and Burke Odland [108] analysed 339 American print and online news articles published between 2013 and 2014, finding that teenagers' relationships with social media were overwhelmingly framed as ‘dysfunctional’ and ‘dangerous’, with teenagers represented as lacking agency and having universally negative experiences. Even where articles *did* mention more positive aspects of use, these were typically counterbalanced by negative commentary, while negatively framed articles were rarely given the same balanced treatment [108]. A similar trend was observed in Denmark, where media coverage predominantly focused on potential negative consequences of adolescent screen time, such as mental health risks, addiction and distraction [110]. Nonetheless, to our knowledge, no such research has been conducted in the UK context.

Given the outlined role of mass media in shaping both public discourse and policy, and the potential impact of coverage on adolescent’s lives and experiences (Fig. 1), there is a pressing need to understand how adolescent social media use is portrayed in UK newspapers. The current study seeks to fill this gap, paying particular attention to the benefits and risks represented. Our specific questions are as follows: (1) how do UK newspapers frame adolescent social media use (positive/negative/neutral)? (2) what are the risks of adolescent social media use represented to be? And (3) what are the benefits of adolescent social media use represented to be?

## Methods

We pre-registered a study protocol, before conducting the initial searches in June 2024 [23]. We conducted a systematic review and content analysis of UK news articles (in print or online) that discussed young people’s use of social media and were published between 2014 and 2024. We selected this timeframe to capture the most recent representations of adolescent social media use, while also affording the capacity to assess potential differences in representations over time. This timeframe is also in line with the widespread adoption of social media among adolescents in the UK (e.g., Ofcom [86]).

### Data corpus

We conducted a systematic search of the Factiva database, a database of global news content that includes archives of UK newspapers across the political spectrum. We searched the database using keywords in a search string to capture articles about adolescent social media use and experiences (see Table 1).

**Table 1:** Search string

Concept	Search terms
Adolescents	("teen"or"youth"or"adolescen*” or"young people"or"child"or “kid” or “boy” or “girl” or “student” or “pupil”)
Social media	("social media"or"social network"or"tiktok"or"snapchat"or"instagram"or “youtube” or “whatsapp” or"facebook"or"screen"or"digital"or"smartphone")^a^

^a^YouTube, WhatsApp, TikTok, Snapchat, Instagram and Facebook are the most commonly used social media platforms among young people in the UK [86]

To ensure the relevancy of the search results and maintain a feasible scope, we restricted the search in several ways: by national newspapers only, subject (social media), region (United Kingdom), language (English), and date (01 January 2014 to 21 June 2024, when the search was conducted). More information regarding these restrictions can be found in the Supplementary file (https://osf.io/qxdyp). In trial searches before implementing these restrictions, we found many articles contained passing references to social media and young people within the body text but were irrelevant for the aims of this study (i.e., did not mention risks or benefits of adolescent social media use). To avoid capturing such tangential mentions, we also restricted the search to headline only. This search identified 397 articles.

**Fig. 2:**
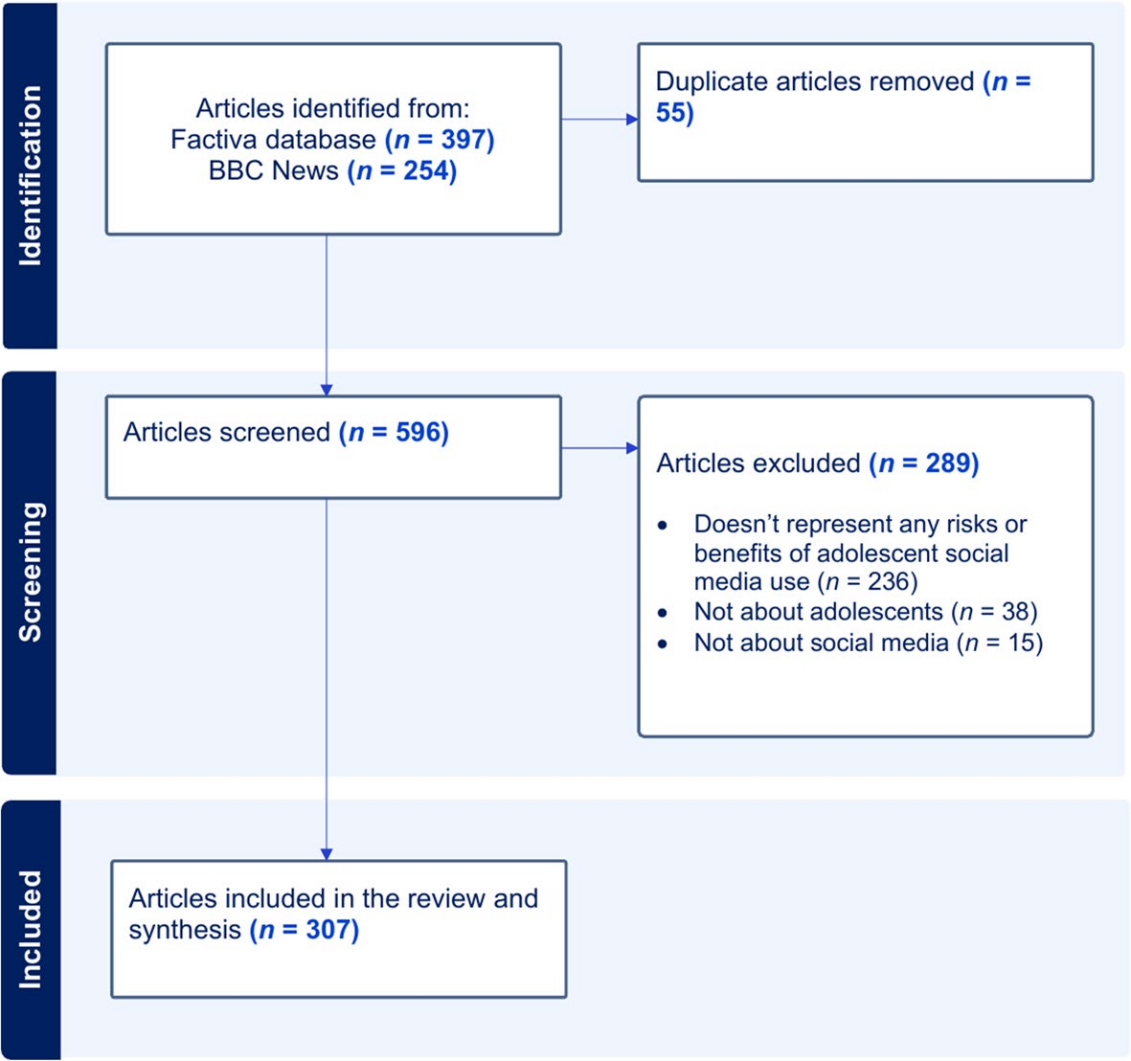
Flowchart of article selection

Although not formally classified as a newspaper, the British Broadcasting Corporation (BBC) website/app is the most commonly used online source for news among adults in the UK [87]. As the UK’s oldest national broadcaster, the BBC is widely regarded as the most trustworthy media outlet by the British public [133]. High levels of trust and widespread consumption mean that the BBC is critical in shaping national discourse. As the Factiva database does not index BBC News, we conducted a separate search of the BBC News website, using Google advanced search. Google advanced search does not allow for as many search terms or restrictions as the Factiva database. As such, we used the search terms we perceived as most relevant, based on our preliminary searches: (“teen” or “child”) and (“social media” or “smartphone”), and restricted the search by domain: https://www.bbc.co.uk/news. This search identified 254 BBC News articles in our specified timeframe (i.e., since 2014). See Fig. 2 for a flowchart illustrating the full article selection process.

### Article screening

JD exported the results from both searches and removed duplicate articles. Where articles appeared extremely similar but were not identical in content (e.g., where an article was published both in print and online, with one version containing more information), only the article with the highest word count was retained. In total, 55 duplicates were identified, and removed, resulting in 596 articles for screening. To be included for analysis, articles had to contain data pertaining to the risks/benefits of adolescent social media use (i.e., address research question 2 and/or 3). We defined adolescence as the ages between 10 and 24 years, inclusive [102], and social media as “any online resource that is designed to facilitate engagement between individuals” [8], p. 63). Based on these definitions, we excluded articles that (a) represented risks/benefits of social media use for non-adolescents (i.e., aged < 10 years or > 24 years), (b) reported more broadly on risks/benefits of the internet or smartphones, with no reference to specific social media platforms or engagement between individuals, or (c) reported generally on adolescent social media use, without discussion of risks/benefits.

All articles were screened by JD and SC for inclusion. Both authors screened a sub-set of the articles together (*n* = 20) to establish the utility of the inclusion/exclusion criteria, before independently screening the remaining articles (*n* = 576). The authors met at regular points to discuss discrepancies. Where they were unsure about the inclusion of particular articles, or could not reach agreement, other authors supported the discussion and made the final judgement. Agreement was substantial (86.1%, κ = 0.72) [61]. In total, 307 articles were included for analysis.

### Data extraction

JD and SC both reviewed the first 40 articles using an a-priori data extraction template. The template included general details about each article including the title, publishing date, and article type (i.e., news, opinion, or newspaper-led research). We noted whether the article used research evidence in its discussion of adolescent social media use and, if so, whether it provided sufficient details to locate the research, and/or any information about the participants, methodology, or limitations of the research. We also recorded any mentions of specific social media platforms in the headline or body text. Finally, we captured broader characteristics about the newspapers the articles were published in, including whether they were tabloids or broadsheets, as well as their political bias (left-wing, left-centre, right-centre, right-wing, least biased) and credibility (high, medium, low), as assessed by https://mediabiasfactcheck.com. We also recorded monthly online readership of the newspapers, as reported by https://pressgazette.co.uk.

After reviewing the first 40 articles independently, JD and SC met to review the extraction and discuss changes that should be made to improve the utility of the template. Changes made at this stage included broadening the criteria for determining whether articles provided sufficient details to locate referenced research (expanding from only hyperlinks to also including authors, publishers, or report titles) and adding the extraction of the type of research cited (academic, grey literature, unclear origin). A full list of changes can be found in V2 of the protocol, and the final extraction template can be found in the Supplementary file.

The full articles were then uploaded to nVivo [69] for analysis. During the analysis, we also extracted the use of quotes (e.g., from young people, parents, law enforcement) to illustrate potential risks and benefits of adolescent social media use. Each quote was coded for: (a) who was being quoted, and (b) the risk or benefit mentioned (see data analysis for further detail). We used an a-priori list of potential quote sources (see protocol V1), which included categories such as ‘parent or other family member’, ‘young person’, and ‘teacher or other education professional’. We refined this list as necessary (see protocol V2). For example, expanding the ‘government official’ category to ‘government or public official’ to better capture the variety of voices represented.

### Data analysis

Before conducting the formal analyses, we conducted a descriptive overview of the coverage regarding adolescent social media use. For example, examining the frequency of publications over time and across different newspapers to identify broader trends in reporting.

To address research question one (*how do UK newspapers frame adolescent social media use (positive/negative/neutral)?)* we conducted a sentiment analysis of article headlines. Sentiment analysis is a technique used to determine the emotional tone or sentiment expressed in text, often classifying it as positive, negative, or neutral [94]. We focused specifically on headlines since they are people’s first – and, sometimes, only – point of engagement, and play a critical role in shaping initial perceptions [33, 41]. Given the apparent unreliability of automated sentiment analysis tools for news research [13], JD manually categorised each headline as positive, negative, or neutral based on its tone and framing. Headlines that portrayed social media use among adolescents as beneficial, empowering, or having a constructive impact were coded as positive. Conversely, those that focused on risks, dangers, or adverse effects of adolescent social media use were coded as negative. Headlines that neither leaned towards a positive nor negative framing (e.g., simply reported facts or posed questions without evaluative language) were coded as neutral.

To address research questions two (*what are the risks of adolescent social media use represented to be?)* and three (*what are the benefits of adolescent social media use represented to be?)* we conducted a qualitative content analysis [50]. We began by applying a directed approach, using two broad a-priori categories of ‘risks’ and ‘benefits’, derived directly from our research questions (see protocol V2 for full details). Within these categories, we developed a detailed coding framework comprising the following sub-categories: (a) 'negative social media experiences' (7 codes, e.g., exposure to harmful content); (b) 'negative outcomes of social media use' (12 codes, e.g., poor mental health); (c) ‘positive social media experiences’ (5 codes, e.g., form new relationships), and (d) ‘positive outcomes of social media use’ (9 codes, e.g., learning). These sub-categories and codes were informed by existing literature on media representations of adolescent social media use (e.g., [108]), and our own research examining the social media experiences of adolescents in the UK (e.g., [49]). As coding progressed, we also took a conventional approach, inductively generating new categories and refining existing ones to better capture what was presented in the articles [50]. For example, the original ‘grooming’ risk category was refined to the broader category of ‘inappropriate contact with adults’ to encompass a wider range of predatory behaviours.

To ensure rigour in our content analysis, JD and SC both independently coded the first 10% of the articles before meeting to compare their coding decisions. Minor discrepancies (e.g., interpretations of ‘exposure to harmful content’) were discussed in depth until the two authors agreed on the interpretation and application of each code. Once agreement was reached, they divided the remaining articles, and each author independently coded half the corpus, with periodic consistency checks to prevent coding drift. Before writing up the findings, JD reviewed all content within each code to confirm internal consistency, though no recoding was necessary.

Finally, we performed a series of exploratory cross-tabulation analyses to assess if, and how, representations of adolescent social media use varied across different newspapers. This involved examining associations between (1) newspaper type and the sentiment of article headlines; (2) newspaper type and the use of research evidence; (3) newspaper type and the use of quotes; (4) newspaper type and the reporting of risks and/or benefits, and (5) who is quoted and whether the quote focused on risks or benefits. Chi-square analyses were conducted using R, with plots created using the ggplot2 package [128]. For brevity, only significant associations are reported here, with further details of all cross-tabulations available in the OSF Supplementary file.

### Reporting decisions

A large portion of our findings involve sensitive topics, most notably adolescent suicide. Previous research [52, 74] suggests that media reporting on these topics often fails to adhere to established guidelines for ethical and responsible journalism, which caution against sensationalism, stigmatisation, glorification, and gratuitous reporting [32, 101, 130]. Such irresponsible reporting on suicide is not only associated with increases in suicide attempts but can also compound the distress experienced by grieving families, friends, and wider communities [16, 47, 80, 106].

While our research differs from general media reporting, we were acutely aware of the linguistic and narrative power in how we chose to present our findings. To ensure our own reporting does not inadvertently reinforce irresponsible, insensitive, and potentially harmful narratives, we made two key methodological decisions. First, we chose not to include identifiable extracts or quotations from specific articles. Our rationale in this regard was twofold: (1) to avoid singling out individual instances of problematic reporting and instead demonstrate broader patterns of discourse present in our data corpus, and (2) to prevent the potential – unintentional – legitimisation of stigmatising discourse through repetition. Second, we intentionally omitted the names of specific young people and cases mentioned in the original newspaper coverage. Although these details may already be public, we felt the sensationalist and sometimes disrespectful nature of the reports risked compounding harm and wanted to prioritise preserving the dignity of those involved.

## Results

### Characteristics of the articles

The sample comprised 307 articles from 13 different UK news sources. Articles spanned the entire range of the period included (2014–2024), with a sharp increase in publications between 2014 and 2019, followed by a slight decline in recent years. As seen in Table 2, BBC News published almost half the included articles (*n* = 142, 46.3%), followed by the Daily Mail/Mail Online (*n* = 61, 19.9 %), and the Sun/Sun Online (*n* = 29, 9.4%). Broadsheet newspapers and their online counterparts comprised the majority of the sample (*n* = 207, 67.4%), with tabloids accounting for the remainder (*n* = 100, 32.6%). The political leaning of the sources was relatively balanced, with 176 left-leaning sources (including left-centre, 57.3%), and 128 right-leaning sources (including right-centre, 41.7%). Around half the articles came from high credibility sources (*n* = 156, 50.8%). Monthly online readership ranged from BBC News's 2.9 billion monthly readers to the Financial Times's 15 million. Articles were predominantly news articles (*n* = 286, 93.2%), with the remainder being opinion pieces (*n* = 16, 5.2%) or newspaper-led research (*n* = 5, 1.6%), Examples of newspaper-led research include articles where journalists went undercover as adolescents on social media and reported on their experience.

**Table 2:** Characteristics of included articles (*n* = 307)

Newspaper	Number of included articles	Tabloid/Broadsheet	Bias^a^	Credibility^b^	Monthly online readership^c^
BBC News	142 (46.3%)	Broadsheet^d^	Left-Centre	High	2.9 billion
Daily Mail/Mail Online	61 (19.9%)	Tabloid	Right	Low	512.4 million
The Sun/The Sun Online	29 (9.4%)	Tabloid	Right	Medium	282.1 million
The Telegraph/The Telegraph Online/The Sunday Telegraph	20 (6.5%)	Broadsheet	Right	Medium	200.3 million
The Independent/ Independent Online	17 (5.5%)	Broadsheet	Left-Centre	Medium	90.4 million
The Times/Sunday Times/Times Online	13 (4.2%)	Broadsheet	Right-Centre	High	40.4 million
The Guardian	12 (3.9%)	Broadsheet	Left-Centre	Medium	238.4 million
The Daily Express	4 (1.3%)	Tabloid	Right	Medium	127.6 million
Metro	4 (1.3%)	Tabloid	Left-Centre	Medium	54.9 million
i	2 (0.7%)	Broadsheet	Unavailable	Unavailable	Unavailable
Daily Star	1 (0.3%)	Tabloid	Right-Centre	Low	25.3 million
Financial Times	1 (0.3%)	Broadsheet	Least biased	High	15 million
The Daily Mirror	1 (0.3%)	Tabloid	Left-Centre	Medium	166.3 million

^a^ Bias is a representation of political leaning and is taken from Media Bias Fact Check (https://mediabiasfactcheck.com, accessed 15th August 2024)

^b^ Credibility is a representation of how factual the reporting is and is taken from Media Bias Fact Check (https://mediabiasfactcheck.com, accessed 15th August 2024)

^c^ Monthly online readership is taken from Ipsos Iris, published by the Press Gazette (https://pressgazette.co.uk) in July 2024, for the period 1–30 June 2024

^d^ While BBC News is primarily an online and broadcast news source, it has been classified as a broadsheet in this study due to its content depth, formal style, and reputation for quality journalism, which align more closely with characteristics of broadsheets than tabloids

Most headlines (*n* = 201, 65.5%) referred generally to 'social media' or the 'online' world,^1^ while the remainder (*n* = 106, 34.5%) referenced specific platforms. Among those, Facebook (*n* = 42 of 106, 39.6%), Instagram (*n* = 36 of 106, 34.0%), and TikTok (*n* = 19 of 106, 17.9%) were the most referenced. Three-quarters (*n* = 233, 75.9%) of articles mentioned specific platforms within the body text, most frequently Facebook (*n* = 143 of 233, 61.4%), Instagram (*n* = 143 of 233, 61.4%) and/or Snapchat (*n* = 73 of 233, 31.3%).

### Sentiment of headlines

**Fig. 3:**
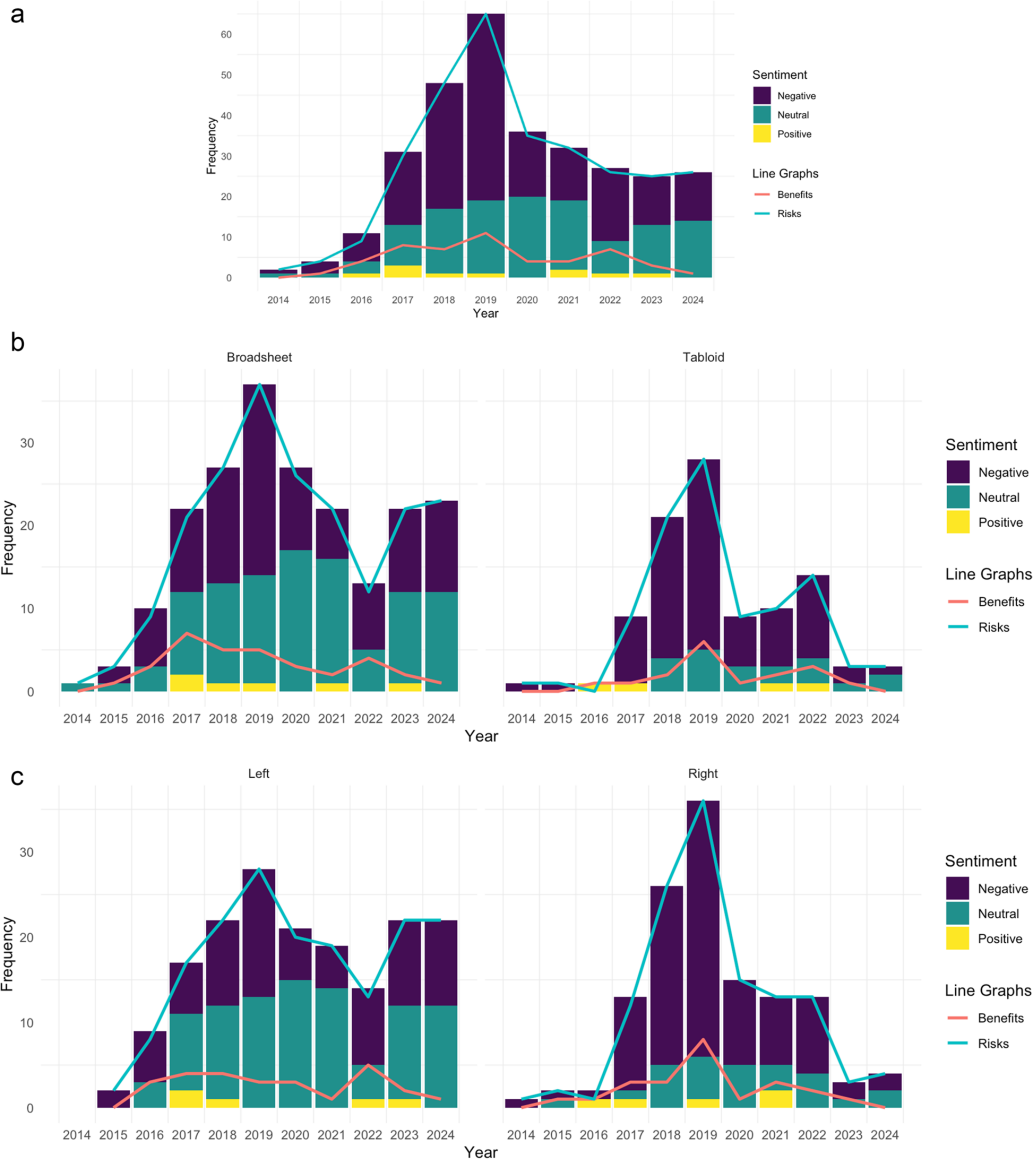
**a** Sentiment and representation of risks and benefits over time, **b** Sentiment and representation of risks and benefits over time: Broadsheet versus tabloid, **c** Sentiment and representation of risks and benefits over time: Left versus right

Over half the headlines were negatively framed (*n* = 177, 57.7%), while almost 40% (*n* = 120, 39.1%) were neutral. Headlines with a positive sentiment were rare (*n* = 10, 3.3%). As seen in Fig. 3a, negative headlines dominated throughout the study period, while articles with positive headlines remained uncommon throughout. A chi-square analysis revealed a statistically significant association between newspaper type (broadsheet vs. tabloid) and headline tone: χ^2^(2, *n* = 307) = 22.73, *p* < 0.001, *V* = 0.272. Broadsheets had significantly fewer negative headlines, and significantly more neutral headlines, while tabloids had significantly more negative headlines, and significantly fewer neutral headlines (see Fig. 3b). A statistically significant association was also found between bias (left-leaning vs. right-leaning) and headline sentiment, χ^2^(2, *n* = 304) = 30.33, *p* < 0.001, *V* = 0.316. Left-leaning newspapers had significantly fewer negative headlines, and significantly more neutral headlines, while right-leaning newspapers had significantly more negative headlines, and significantly fewer neutral headlines (see Fig. 3c).

### Use of research evidence

Less than one-third of the articles (*n* = 96, 31.3%) used research evidence to support their discussion of the potential risks and/or benefits of adolescent social media use. Of those, 13 articles referenced more than one research output, resulting in a total of 122 relevant research references across the entire corpus. Grey literature (e.g., charity-led research, government reports) was drawn upon the most (*n* = 73 of 122, 59.8%), followed by academic literature (*n* = 38 of 122, 31.2%). A minority of references (*n* = 11 of 122, 9.0%) drew upon literature with an unclear origin. These references made vague claims about research findings, without specifying their sources. For example, one article claimed almost half the young people in a study said social media negatively impacted their mood or mental health but failed to provide any information on the source of these findings. Reporting of research details was inconsistent, with 78 (of 122, 63.9%) references including details regarding the sample, 63 (of 122, 51.6%) including details of the research methodology, and only 22 (of 122, 18.0%) mentioning any limitations or caveats of the research. Less than half (*n* = 57 of 122, 46.7%) provided sufficient details about where to access the research (e.g., a report title, author, or hyperlink).

### Use of quotes

Quotes were used in 215 articles (70.0%) to illustrate potential risks and/or benefits of adolescent social media use. The most frequently quoted sources included government/public officials (*n* = 59 of 215, 27.4%), charity/not-for-profit representatives (*n* = 52 of 215, 24.2%), and parents or other family members (*n* = 50 of 215, 23.3%). Young people’s voices were represented in 34 articles (of 215, 15.8%), followed by medical professionals (*n* = 23 of 215, 10.7%) and police and law enforcement (*n* = 23 of 215, 10.7%). Researchers (*n* = 22 of 215, 10.2%), social media representatives (*n* = 15 of 215, 7.0%), and teachers and other education professionals (*n* = 13 of 215, 6.0%) were also drawn upon, albeit less frequently. A small number of articles (*n* = 12 of 215, 5.6%) included quotes from ‘other’ sources, such as celebrities.

There was a statistically significant association between newspaper type (broadsheet vs. tabloid) and the sources quoted: χ^2^ (9, *n* = 327) = 20.04, *p* = 0.017, *V* = 0.248. Broadsheet articles quoted government/public officials, police or legal experts, researchers, and young people more often, and social media representatives and ‘other’ sources less often. Tabloids, on the other hand, quoted parents or family members, social media representatives, and ‘other’ sources more often, while quoting government/public officials and police or legal experts less often. A statistically significant association between newspaper bias (left-leaning vs. right-leaning) and quoted sources was also found: χ^2^(9, *n* = 327) = 22.81, *p* < 0.001, *V* = 0.266. Left-leaning newspapers quoted government/public officials, police or legal experts, researchers, and young people more frequently, and parents or family members, medical professionals, and ‘other’ sources less frequently. In contrast, right-leaning papers quoted medical professionals, parents or family members, and ‘other’ sources more frequently, while quoting government/public officials, police or legal experts, and researchers less frequently.

### Representation of risks and benefits

Almost all articles (*n* = 302 of 307, 98.4%) mentioned at least one risk associated with young people’s use of social media (see Table 3), while only 16.3% (*n* = 50) discussed any potential benefits (see Table 4). Discussion of risks was more common than discussion of benefits throughout the entire study period (see Fig. 3a). Most articles *only* discussed risks (*n* = 257, 83.7%), with a small number (*n* = 45, 14.7%) mentioning both risks and benefits, and only 1.6% (*n* = 5) discussing benefits alone. In discussing these risks and benefits, the articles drew on a variety of sources. A statistically significant association was found between who was quoted and the nature of the quoted content (risk vs. benefit): χ^2^(9, *n* = 448) = 93.86, *p* < 0.001, *V* = 0.458. Quotes from charity representatives, government officials, and family members were significantly more likely to represent risks. Conversely, researchers, social media representatives, and young people were significantly more likely to be quoted on benefits.

**Table 3:** Summary of all risks represented (*n* = 302)

Risk	N (%)
Risks – experiences and activities (***n* = 224, 74.2%)**	
Access/exposure to harmful content	109 (36.1%)
Online conflict (including cyberbullying)	78 (25.8%)
Inappropriate contact with adults	67 (22.2%)
Exposure to misinformation	25 (8.3%)
Access to and/or creation of extreme content	20 (6.6%)
Privacy concerns	16 (5.3%)
Sexting and revenge porn	14 (4.6%)
Social media is an echo chamber	4 (1.3%)
Stalking	3 (1.0%)
Risks – outcomes of social media use (***n* = 199, 65.9%)**	
Poor mental health	157 (52.0%)
Addiction	46 (15.2%)
Poor physical health (e.g., poor diet, sleep)	26 (8.6%)
Engaging in anti-social or illegal activities	13 (4.3%)
Engaging in risky behaviour	13 (4.3%)
Distraction	8 (2.7%)
Damaged relationships	7 (2.3%)
Poor finances	7 (2.3%)
Reduced social skills	6 (2.0%)
Regretting social media activity/posts	2 (0.7%)
Poor educational outcomes	2 (0.7%)

**Table 4:** Summary of all benefits represented (*n* = 50)

Benefit	N (%)
Benefits – experiences and activities (***n* = 43, 86.0%)**	
Social connection and support	39 (78.0%)
Self-expression	10 (20.0%)
Positive messaging	4 (8.0%)
Coping	3 (6.0%)
Representation	3 (6.0%)
Benefits – outcomes of social media use (***n* = 29, 58.0%)**	
Benefits for mental health	15 (30.0%)
Making money and/or social media as a career	8 (16.0%)
Improved confidence	6 (12.0%)
Learning	5 (10.0%)
Improved social skills	3 (6.0%)
Creativity	2 (4.0%)
Pro-social behaviour	1 (2.0%)

#### What are the risks of adolescent social media use represented to be?

In total, we identified 20 different risks associated with adolescent social media use, including potential negative experiences while using social media, as well as potential negative outcomes associated with social media use (see Table 3). While a broad range of risks were mentioned, four key concerns dominated the coverage: negative consequences for mental health, exposure to harmful content, online conflict, and inappropriate contact with adults. These risks were not only the most frequently discussed but were also often framed as being interconnected, with each risk compounding another. The following sections provide an in-depth examination of how these four key risks were represented.

##### **Negative consequences for mental health**

The potential for social media use to negatively impact mental health was the most prevalent concern, featuring in over half the articles that discussed risks (*n* = 157 of 302, 52.0%). The articles varied in how they framed the relationship between social media use and mental health outcomes. Some made explicit causal claims, suggesting that society has ‘long known’ about the ‘damaging’ effects of social media on young people’s mental health. Other articles linked social media use to poor mental health, without explicitly attributing causality. A minority of articles acknowledged ongoing academic debate regarding causality, often using quotes from researchers and/or charity representatives.

Within the context of risks for mental health, suicide was identified as a major concern. Of the articles that identified poor mental health as a risk, almost half discussed suicidality and/or death by suicide (*n* = 73 of 157, 46.5%), representing almost one in four of all the included articles (*n* = 73 of 307, 23.8%). These articles frequently referenced high-profile cases, with more than two-thirds (*n* = 49 of 73, 67.1%) referencing the case of a specific, named teenager whose suicide was linked to social media by a coroner. Beyond suicide-related concerns, articles addressed a range of other potential mental health risks. General references to mental health and wellbeing, without specification of particular conditions, appeared in 57 articles (of 157 36.3%). Where articles were more specific, they highlighted concerns about self-esteem and body image (*n* = 24 of 157, 15.3%), anxiety (*n* = 23 of 157, 14.7%), depression (*n* = 22 of 157, 14.0%) and self-harm (*n* = 15 of 157, 9.6%). Other identified risks for mental health included loneliness (*n* = 11 of 157, 7.0%), fear of missing out (*n* = 9 of 157, 5.7%), general low mood (*n* = 9 of 157, 5.7%), eating disorders (*n* = 6 of 157, 3.8%) and reduced life satisfaction (*n* = 3 of 157, 1.9%).

In one-quarter (*n* = 40 of 157, 25.5%) of the cases, the article did not specify the mechanism through which social media was purported to negatively impact adolescent mental health. Where mechanisms *were* specified, exposure to harmful content, such as material promoting self-harm or eating disorders, was the most frequently cited (*n* = 56 of 157, 35.7%). Behavioural mechanisms included excessive time spent on social media (*n* = 25 of 157, 15.9%) and social media addiction (*n* = 5 of 157, 3.2%). Social mechanisms encompassed online conflict (e.g., cyberbullying) (*n* = 16 of 157, 10.2%), self-comparison (*n* = 14 of 157, 8.9%), and pressures of maintaining an online presence (*n* = 12 of 157, 7.6%). Less frequently mentioned mechanisms included displacement of healthier activities (*n* = 4 of 157, 2.6%), inappropriate contact with adults (*n* = 3 of 157, 1.9%), the need for validation (*n* = 2 of 157, 1.3%), engagement in risky behaviour (*n* = 1 of 157, 0.6%), and passive social media use (*n* = 1 of 157, 0.6%).

##### **Exposure to harmful content**

Articles (*n* = 109 of 302, 36.1%) commonly highlighted the risk of exposure to harmful content. Here too, portrayals varied. Some articles referred vaguely to ‘dangerous’ or ‘harmful’ content, while others provided specific examples. For example, several articles quoted a coroner who spoke of the ‘disturbing’ and ‘graphic’ content one teenager who died by suicide accessed before their death, ruling that it should not have been available for any child to see. The perceived degree of user agency in encountering such content also varied in coverage. For example, some articles described young people as being ‘bombarded’ with harmful content, including content about suicide. Others suggested some young people may actively seek out such content. For example, a journalist who went undercover as a teenager on social media explained that, despite some platforms' attempts to block harmful material, it remains easily accessible to those looking for it on other platforms.

##### **Online conflict**

Online conflict was the third most frequently mentioned risk, appearing in 78 (of 302, 25.8%) articles. Many references to online conflict were vague, referring generally to ‘cyberbullying’ without providing additional detail. Those that were more specific tended to focus on lower-level online conflict, such as ‘nasty’ comments being left on one’s posts, while a minority of articles highlighted more extreme cases. For example, one article detailed the story of a teenager who was murdered following a dispute on social media. Although, in the article, the teenager’s father suggested social media was not guilty of the murder, the article’s headline attributed blame to social media.

##### **Inappropriate contact with adults**

Inappropriate contact with adults was mentioned in almost one-quarter of the articles mentioning potential risks (*n* = 67 of 302, 22.2%). The degree to which social media platforms were held responsible for facilitating such behaviour varied. Some articles suggested social media play an active role, citing instances where naked men were suggested as friends to teenagers. Other articles portrayed platforms as simply being tools misused by predators.

#### What are the benefits of adolescent social media use represented to be?

We identified 12 different benefits that were reported to be associated with adolescent social media use (see Table 4). As with the presentation of risks, these benefits included both potential positive experiences while using social media as well as potential positive outcomes of social media use. Most commonly, articles discussed the potential for social connection and support, positive impacts on mental health, and self-expression. The following sections examine how these key benefits were represented.

##### **Social connection and support**

The most commonly discussed benefit was social media’s ability to facilitate social connection and support, which appeared in over three-quarters of the articles discussing potential benefits (*n* = 39 of 50, 78.0%). The portrayal of social connection varied in nature and specificity. Some articles spoke generally about social media allowing young people to ‘build communities’ and ‘keep up friendships’, while other articles highlighted more specific ways that groups of young people benefit from connecting with like-minded peers. For example, a young person interviewed in one article explained how they were able to connect and relate with other LGBT people they found online, something they were not able to do in ‘real’ life.

##### **Positive impacts on mental health**

Some articles (*n* = 15 of 50, 30.0%) presented the potential for social media to have positive impacts on mental health, though these perspectives were often counterbalanced with potential risks. For example, one article suggested social media may provide a ‘temporary’ improvement for wellbeing for certain young people, but that the longer-term effects are negative. Another article suggested that social media has ‘the potential’ to contribute to good mental health, but there are significant risks that must be addressed if this is the case. Other articles suggested mental health benefits may be limited to certain platforms, most commonly YouTube.

##### **Self-expression**

Self-expression was identified as another key benefit of adolescent social media use (*n *= 10 of 50, 20.0%). Most of these articles made general claims about social media promoting self-expression, though some did highlight specific platforms that may be particularly supportive of this benefit. In particular, TikTok was noted as a popular avenue for young people to express themselves in ways they might not feel comfortable doing offline.

## Discussion

This study critically examined how UK newspapers portray adolescent social media use, with particular reference to the risks and benefits represented. We identified a dominant narrative of panic that frames social media as fundamentally threatening for adolescents. Headlines were frequently negatively framed (RQ1), and the overall discourse focused predominantly on risks, including (but not limited to) compromised mental health, exposure to harmful content, and online conflict (RQ2). These concerns were amplified through provocative headlines, quotes from government officials and charities, and limited engagement with academic research evidence. Although benefits like social connection and self-expression (RQ3) were occasionally acknowledged – often by young people themselves – they remained subsumed by an overarching narrative of caution and risk [108]. In the following sections, we critically examine our findings in relation to existing research, and make recommendations for future research, policy, and practice.

### The representation of social media use and adolescent mental health

Newspaper portrayals of social media's impact on adolescent mental health consistently emphasised potential harms, with some articles suggesting causal links between social media use and poor mental health. Given that not all articles were based on empirical evidence, and of those that were, the majority failed to provide sufficient details of the studies reported, this framing cannot be substantiated. More generally, this is inconsistent with the current empirical evidence base. Indeed, cross-sectional studies, which are unable to establish causal relationships, have dominated much of the research landscape to date [89], and longitudinal designs frequently find no evidence for a direct relationship between social media use and poor adolescent mental health [3, 21, 37, 96]. Some studies even suggest the reverse – that poor mental health may predict increased social media use – and/or that any predictive effects are limited to specific subgroups [11, 93, 98, 103]. Notably, in one study which explored the interrelationships of social media use and other factors such as bullying, family support, and academic dissatisfaction, social media ranked among the least influential predictors in the system [93]. This more nuanced understanding is further supported by Yang and Feng’s [131] meta-analysis of 73 studies which found no substantive support for direct associations between social media use and subjective well-being, instead suggesting that psychological factors like perceived social support and self-esteem had considerably stronger effects. Such crucial nuances in the impact of social media use on adolescent mental health were largely absent in the articles reviewed.

The tendency to overstate and/or oversimplify the relationship between social media use and adolescent mental health was also reflected in the specific mental health risks identified. Nearly 25% of all the included articles mentioned suicidality and/or suicide in their discussion of adolescent social media use. This is despite evidence suggesting that social media use shows only weak, if any, correlations with self-reported mental health and wellbeing among adolescents [90, 124]. The focus on suicide in relation to adolescent social media use appears especially disproportionate given that research highlights much stronger associations between structural social factors – such as income inequality and fatherlessness – and poor mental health outcomes, than social media and poor mental health outcomes (Ferguson, 2025).

Beyond the lack of empirical justification for the focus on suicide as a potential consequence of adolescent social media use, the way this was discussed raised serious ethical concerns. Indeed, these articles often used sensationalist language, framing the deaths as being a direct result of the young person being ‘hooked’ on social media, or their ‘quest’ to get likes and validation. This reductive framing obscures the complex and multifaceted contributors to adolescent suicide – such as bullying, family conflict, trauma, pre-existing mental health conditions, and systemic factors like socioeconomic disadvantage, discrimination, and barriers to mental health care – that are well-documented in empirical research [1, 45, 60, 65, 72, 97, 114, 132]. Some articles also employed dehumanising language when talking about the impact of social media use on adolescent mental health, including multiple articles that referred to a young person who died by suicide as 'suicide girl'. Others went further, explicitly mentioning suicide methods and/or providing graphic details of the circumstances surrounding the young person’s death in the article headline or body text. Such practices are not only insensitive and disrespectful to the young people and families involved, but also directly contradict guidelines on responsible suicide reporting, which emphasise the importance of avoiding sensationalism, stigmatisation, glorification, and gratuitous reporting [32, 101, 130]. Sadly, this is not an isolated issue – research has repeatedly shown that British media reporting on suicide consistently fails to meet ethical journalistic standards [52, 74].

While the loss of young lives to suicide is an incredibly serious and important topic that unquestionably warrants media attention, our findings highlight widespread problematic reporting practices that are inaccurate, insensitive and potentially harmful. Indeed, some evidence suggests that irresponsible media reporting may contribute to increased suicide attempts (the Werther effect) and exacerbate the distress of grieving families and friends [16, 47, 80, 106]. Conversely, responsible reporting may be positive (the Papageno effect), reducing suicidal ideation and stimulating help-seeking behaviours among people considering suicide [78, 79, 81, 113]. Empirical evidence regarding these effects varies: most studies point toward the Werther effect (*n* = 69 of 98, 70.4%) or the Papageno effect (*n* = 4 of 98, 4.1%) while others show no effect (*n* = 16 of 98, 16.3%) or conflicting results (*n* = 9 of 98, 9.2%) [30]. Nonetheless, experts broadly agree that sensationalist reporting should be avoided [30]. As such, we posit that the identified sensationalist reporting on this issue represents a critical missed opportunity to foster a more constructive public dialogue – one that acknowledges the complex realities of adolescent suicide, and the preventative measures needed to address it. Moving forward, journalists must seek to adhere to established guidelines on responsible reporting of suicide and mental health, ensuring their coverage is evidence-based, compassionate, and aligned with ethical standards.

Discussions of the potential benefits of adolescent social media use, such as social connection, were not only rare but often counterbalanced by risks [108]. It may be that this framing reflects an underlying truth – that social media poses significant risks to adolescents and very few benefits. However, in the absence of a mature evidence base, it is near impossible to make judgements about this balance. It is also important to acknowledge that young people themselves often point toward ‘good’ sides of social media, alongside potential negatives [49, 83, 84, 125]. This discrepancy between media narratives and young people's lived experiences raises questions about whether the risks are being overemphasised at the expense of exploring potential benefits. This same problem has already been illustrated in the case of violent video games, with negative findings – rather than large effect sizes or high methodological quality – increasing the likelihood of receiving news media coverage, suggesting this is a perennial problem for news media [20].

At the same time, other reviews have highlighted the relative lack of research attention paid toward examining potential positive aspects of adolescent social media use [105]. This lack of research attention to benefits may reflect a broader pattern shaped by moral panic, in which heightened public concern over perceived harms fuels demand for research into negative outcomes [36, 42]. This, in turn, directs funding and scholarly interest toward adverse effects, reinforcing the cycle and potentially overlooking more balanced or beneficial dimensions of adolescent social media use [36, 42]. Ultimately, however, this imbalance may limit our understanding of how social media can be leveraged as a tool to support adolescent development and wellbeing, leaving a critical gap in both research and public discourse.

This limited and often one-sided discourse also overlooks potential variation in adolescents’ experiences with social media, presenting a blanket narrative that fails to capture their diverse realities. For example, marginalised groups such as young people who identify as LGBTQ + report finding social media a vital source of community support and identity affirmation, which can positively impact their mental health [5, 35]. Conversely, victims of cyberbullying may experience particularly negative effects [12, 51, 63, 120].

### Potential implications of such biased media reporting

The potential implications of the outlined biased media reporting are far-reaching. At the microsystem level, the pervasive media focus on the potential harms of adolescent social media use may contribute to increased concern among parents and caregivers. In response to such concerns, some parents may turn to more restrictive digital parenting strategies. Evidence of this phenomenon can be seen with the rise of movements like “smartphone-free childhood” which promote limiting or even eliminating digital devices from children's lives to protect them from perceived harms. Yet, evidence regarding whether restrictive digital parenting approaches are effective in reducing harm remains mixed (see [123] for a review). Within the mesosystem, schools may follow a similar pattern, implementing restrictive mobile phone policies in line with dominant media narratives. However, the limited available evidence suggests these policies are not effective [46]. As such, while biased media narratives may push parents and schools toward restrictive practices, these such approaches may not always be effective and may, in some cases, exacerbate the very issues they seek to address.

Biased media reporting may also influence the attitudes and practices of mental health professionals, situated in the exosystem. In the UK, studies suggest clinicians rarely engage young people in meaningful discussions about their online lives, and when they do, such interactions are often perceived as judgmental or overly simplistic [10, 29, 100]. Practitioners have also been reported to recommend complete withdrawal or blocking strategies in response to negative online experiences, approaches that young people say increase their isolation and disconnect them from supportive online communities [29]. These challenges are compounded by a lack of training and a widely expressed need among practitioners for guidance on how to effectively discuss social media use with young people [29]. Without this support, well-intentioned adults may risk missing opportunities for collaborative dialogue and unintentionally undermine the trust essential to effective therapeutic relationships.

These biased media narratives may also impact young people’s own perceptions and behaviours. Adults may inadvertently pass their concerns on to young people, leading them to internalise negative beliefs about their own social media use [48]. Such negative mindsets around social media use are associated with poorer psychological wellbeing [62] and may influence how adolescents evaluate and manage their own social media use, as well as how they evaluate efforts to reduce their use. For example, anecdotally, some adolescents report that engaging in a digital detox (i.e., abstaining from using digital devices and social media or reducing time spent on social media) has a positive impact on their sleep, concentration, and mood (e.g., Johnson [53],). However, this is not entirely surprising, given the pervasive message that social media is inherently harmful, and that such actions are necessary.

This pattern is reminiscent of concerns surrounding mental health awareness campaigns, which may cause young people to identify low-level symptomology as indicative of disorder, and/or create a self-fulfilling prophecy through which young people do develop clinical symptomology [39]. Here, it is possible that consistently telling young people that social media harms their mental health might itself increase their anxiety about their social media use and/or make them more likely to attribute negative emotions and psychological experiences to their social media use. This is problematic because it could lead to young people experiencing unnecessary feelings of guilt, make them less likely to seek help, and undermine their ability to develop balanced, healthy relationships with social media. Moreover, this creates potential challenges for researchers, as it becomes increasingly difficult to differentiate between what are genuine effects of reduced social media use, and what are perceived effects influenced by one’s expectations and mindset. The potential for these unintended effects may be an important avenue for future research.

Finally, at the macrosystem level, these biased media narratives may shape political discourse and policymaking. In line with media framing, political parties in the UK have taken increasingly firmer stances on the risks associated with its use. The Conservative Party's 2024 manifesto pledged urgent consultation on further parental controls and stricter enforcement of the Online Safety Act [19] while Labour similarly spoke of protecting young people from “significant harm online” [59], (p. 103). Globally, restrictive measures are gaining momentum. For example, in November 2024, the Australian Government passed legislation to ban social media for people under 16 [95], with the Senator stating that “social media … is … contributing to a growing crisis in our children’s mental health” [58], p. 5472). In the United States, the Surgeon General has spoken of the need for robust protections against the harms of social media [116], and several states have explored and/or implemented legislation to further regulate social media for young people (e.g., Florida [38], Utah [117]).

While some of these measures respond to genuine risks (e.g. exposure to harmful content), others, especially those that are more restrictive (e.g. complete bans), risk oversimplifying the complex relationship between social media and adolescent mental health. Such policies may exacerbate existing fears among parents and carers while marginalising young people's voices in shaping discussions about their digital lives. Indeed, blanket restrictions ignore the reality that social media is where most young people conduct a significant portion of their socialisation, with research suggesting these platforms can actually strengthen adolescent relationships when used appropriately [126]. These restrictive approaches also risk reinforcing the notion that young people are incapable of managing technology responsibly, a narrative that contrasts with evidence that young people are not only capable of navigating online risks but are actively engaged in managing their privacy and safety on social media platforms [22, 68, 70, 73]. Recognising young people’s capacity to manage risks does not, however, negate their need for support in building resilience, autonomy, and the skills necessary to navigate difficulties online [6]. Future initiatives should seek to find an appropriate balance between safeguarding young people from genuine harm and empowering them to develop the knowledge and skills required to navigate digital spaces safely.

### Limitations

This study is not without limitations. First, the findings of this study are necessarily limited to the UK context and may not be directly generalisable to other countries. While similarities were found with analyses conducted in other Western nations [108, 110], differences in cultural, political, and regulatory environments could lead to distinct media narratives and public perceptions in other regions. As such, caution should be taken when applying these findings to other contexts, and further research could explore how media narratives surrounding social media and mental health differ across diverse global settings. Second, the study period (2014–2024) coincides with several high-profile adolescent suicides, as well as high-profile legislative events, such as the publication of the Online Harms White Paper in 2019 and the Online Safety Act in 2023 [24, 25]. Indeed, media coverage peaked in 2019, coinciding with the release of the Online Harms White Paper. While these events likely influenced the tone and framing of the media coverage analysed, it is also important to recognise that media narratives are likely to be shaped by multiple systems (Fig. 1). Third, the focus of this study was limited to newspaper representations, which may not reflect discourse in other media formats (e.g., news broadcasts) which are subject to government regulation. Fourth, we did not examine reader comments or public responses to the articles. These forms of engagement could offer valuable insights into how the narratives were received, interpreted, and acted upon by the public. This may be a fruitful avenue for future research. Finally, it is important to acknowledge that the analysis presented here is based on our own interpretations of the news coverage, which are inevitably shaped by our own beliefs, experiences, and contexts, as well as our position as adults. Young people’s readings of these articles may have yielded different results. For example, we categorised headlines that posed questions (e.g., is social media causing depression?) as neutral. Yet, young people may not interpret headlines in this same way. Similarly, young people may have had different interpretations of the risks/benefits represented. Future research would benefit from including adolescents as collaborators in the process of interpreting and critiquing news narratives, as this could offer a more nuanced understanding of how media content is received and understood by the very demographic being discussed.

## Conclusion

Over the past decade, British news articles have consistently painted social media as a dangerous influence on adolescents, often neglecting any potential benefits. By emphasising risks without fully exploring the nuances of adolescent’s social media use, these narratives contribute to a skewed public perception that may shape ineffective policies and interventions. Such narratives may also increase the divide between adolescents and adults, making open conversations and mutual trust more difficult to foster. Future research, clinical practice, and policy development must seek to adopt a more balanced and evidence-based approach, considering both the risks and potential benefits of social media use in adolescents' lives.

## Data Availability

The dataset supporting the conclusions of this article is available to access at: https://osf.io/qxdyp.

## Footnotes

1 Note: BBC News articles were included in the dataset via Google advanced search, which does not allow search restriction to headline only. Consequently, some articles with broad headlines referencing the ‘online’ world were captured. These articles were only included in the analysis if the body text specifically discussed social media, in accordance with our inclusion criteria.
